# Factors influencing digital literacy among the 9185 elderly in South Korea: A machine learning approach

**DOI:** 10.1097/MD.0000000000046761

**Published:** 2026-01-02

**Authors:** Haewon Byeon

**Affiliations:** a Worker’s Care & Digital Health Lab, Department of Future Technology, Korea University of Technology and Education, Cheonan, South Korea.

**Keywords:** CatBoost, digital literacy, elderly individuals, logistic regression, machine learning

## Abstract

This study aims to identify key factors influencing digital literacy among the elderly individuals in South Korea using advanced machine learning techniques, focusing on enhancing their quality of life and social participation. Data from the 2018 to 2019 National Survey of Older Koreans were utilized, encompassing responses from 9185 individuals aged 65 and above. Digital literacy was assessed through the ability to use various digital devices and applications. Predictors included demographic, socioeconomic, health, and social support variables. CatBoost, a gradient boosting algorithm, was employed for feature selection and importance ranking, followed by logistic regression analysis to determine the relationships between these predictors and digital literacy levels. The analysis revealed that higher education levels, employment status, economic status, social participation, subjective health, and informal support positively influenced digital literacy among the elderly individuals. Conversely, age and depression were negatively associated with digital literacy. The CatBoost model demonstrated superior performance in identifying significant predictors, with education level emerging as the most influential factor, followed by age and employment status. The findings highlight the multifaceted nature of digital literacy among the elderly individuals, emphasizing the importance of educational attainment, social engagement, and economic conditions. Targeted interventions addressing these factors can effectively bridge the digital divide, ensuring that the elderly population can fully participate in the digital age and enhance their overall well-being.

## 1. Introduction

In the rapidly evolving landscape of information and communication technology, digital literacy has emerged as a crucial competency for navigating the interconnected world we live in. Digital literacy encompasses the ability to access, understand, and use digital devices and information, which is essential for participating in today’s digital society. As the Fourth Industrial Revolution progresses, many policies are increasingly focused on promoting digitalization and ensuring that all citizens can benefit from technological advancements. Despite these efforts, a persistent gap remains, particularly among older adults, limiting their engagement in digital environments.^[1]^

Elderly individuals are one of the most vulnerable groups when it comes to digital exclusion. The digital divide not only reduces their ability to use digital devices but also negatively impacts their quality of life, economic activities, and social participation. Previous research has highlighted the correlation between digital literacy and various aspects of life, such as social engagement and overall well-being. For instance, studies have shown that higher levels of digital literacy among the elderly individuals are associated with increased social participation and satisfaction. Despite the growing body of literature on digital literacy, there is still a lack of comprehensive studies that focus on the factors influencing digital literacy among the elderly individuals and the impact of these factors on their social activities.

One of the main limitations of previous research is the focus on comparing the digital literacy levels of the elderly individuals with those of the general adult population, rather than examining the specific factors that influence digital literacy within the elderly group itself. This comparative approach often overlooks the internal heterogeneity among older adults, such as differences in health status, educational background, and social integration, which may significantly affect their digital skills. As a result, policies derived from such studies may fail to address the nuanced needs of distinct subgroups within the elderly population, leading to ineffective or misdirected interventions. This approach masks the diverse challenges and capabilities that exist within the aging population.^[2]^ Additionally, many studies have relied on self-reported measures of digital literacy, which may not accurately reflect the actual digital competencies of the elderly.^[3]^ There is a need for more objective and comprehensive assessments of digital literacy that take into account the diverse experiences and capabilities of the elderly population. For instance, the digital literacy of the elderly population lags behind that of the younger generation, a disparity more profound in regions with higher levels of enterprise digitalization.^[2]^ This gap underscores the necessity for targeted interventions that consider the elderly’s unique learning needs and the technological advancements in their environment.

Moreover, basic digital literacy expectations often exceed what is reasonable for older adults without prior digital experience, underscoring the challenges inherent in acquiring even seemingly simple skills such as turning on a device or downloading apps.^[3]^ Programs such as the Digital Academy for Seniors have been significant in developing digital resilience and improving the life quality of Indonesian seniors through a nonformal learning approach, emphasizing the importance of tailored educational initiatives.^[4]^ The emergence of COVID-19 has catalyzed a significant leap from blended learning to digital literacy among the elderly, demonstrating substantial improvements in digital competency despite the pandemic.^[5]^

Furthermore, an educational program combining IT and humanities demonstrated significant improvements in both the recognition and behavioral aspects of digital literacy among the elderly, reinforcing the critical role of comprehensive educational strategies.^[6]^ Research also indicates that digital literacy in the elderly has potential impacts on their psychological well-being and life satisfaction, highlighting the broader implications of digital competency beyond mere technological ability.^[7]^ Finally, experiences since 2009 in digital literacy workshops for the elderly have shown their interest in engaging with the university environment and sharing experiences as a way to promote digital literacy, showcasing the potential for community-driven initiatives in enhancing digital skills.^[8]^

In light of these limitations, this study aims to identify the key factors influencing digital literacy among the elderly in South Korea using advanced machine learning techniques. South Korea provides a particularly compelling context for this investigation. As one of the most rapidly aging countries globally, it is projected to become a “super-aged” society by 2025, with over 20% of the population aged 65 and above.^[6]^ Simultaneously, South Korea is a global leader in digital innovation and information and communication technology infrastructure, including high internet penetration, widespread smartphone use, and e-government services. This stark contrast between technological advancement and the increasing risk of digital exclusion among older adults makes the South Korean elderly population an essential focus for digital literacy research.

Understanding the predictors of digital literacy in this setting are not only socially urgent but also globally informative, offering insights for other countries experiencing similar demographic and technological transitions.

Moreover, the complex and multidimensional nature of digital literacy, encompassing education, health, economic, and social variables, requires analytical approaches capable of modeling nonlinear relationships and high-dimensional data structures. Traditional statistical methods often fall short in capturing such complexity. Thus, this study employs machine learning techniques, which offer a flexible, data-driven framework to uncover hidden patterns. In particular, CatBoost was chosen for its superior handling of categorical variables, resistance to overfitting, and ability to yield interpretable feature importance through Shapley Additive Explanations (SHAP) values. These characteristics make CatBoost especially well-suited to large-scale, survey-based analyses such as this. This study aimed to develop a logistic regression model that leverages the hyperparameter tuning capabilities of CatBoost to identify the most important predictors of digital literacy and inform policymakers, social service providers, and educators on areas where targeted interventions are most needed.

## 2. Methods

### 2.1. Data source and participants

This study utilized data from the 2018 to 2019 National Survey of Older Koreans, conducted by the Ministry of Health and Welfare and the Korea Institute for Health and Social Affairs. The dataset includes responses from 9185 elderly individuals aged 65 and above. The survey provides comprehensive information on various aspects of the elderly population, including their digital literacy, social activities, health status, and economic conditions.

The data collection process involved face-to-face interviews conducted by trained interviewers using standardized questionnaires. These interviews were designed to gather detailed information on the respondents’ demographic characteristics, digital literacy, social participation, health status, and economic conditions. The survey employed a stratified 2-stage cluster sampling method to ensure the representativeness of the sample. In the first stage, strata were defined based on key demographic and geographic characteristics, including urban versus rural residence, administrative region (metropolitan vs provincial), and age subgroups (65–69, 70–74, 75–79, 80+). These strata were selected to reflect Korea’s aging population structure and to ensure proportional representation across residential types and age groups. In the second stage, enumeration districts within each stratum were randomly selected as primary sampling units, followed by systematic sampling of households.

As this study is based on secondary analysis of national epidemiological data, it was exempted from Institutional Review Board (IRB) approval. The exemption was granted by the IRB of INJE University (IRB No. INJE/2021_99_HR). The original data collection was conducted by the Korea Institute for Health and Social Affairs, following national research ethics protocols. All participants provided written informed consent prior to the interviews, and personal identifiers were removed to ensure anonymity. The dataset made available for secondary analysis was fully anonymized and did not include any sensitive personal information.

### 2.2. Variables measurement and definition

#### 2.2.1. Digital literacy

Digital literacy was assessed based on the respondents’ ability to use various digital devices and applications, such as smartphones, tablets, and internet-enabled TVs. The measurement included 10 specific activities: receiving text messages, sending text messages, searching for and viewing information such as news and weather, taking photos and videos, listening to music (e.g., MP3), playing games, watching videos, using social network services, online shopping, and other digital activities. For each of these 10 items, respondents were asked to indicate whether they could perform the activity using a binary scale (0 for “No” and 1 for “Yes”). The total digital literacy score for each respondent was calculated by summing the number of activities they responded “Yes”, resulting in a score ranging from 0 to 10. To ensure the validity of this aggregated scale, an internal consistency check was conducted using Cronbach alpha. The resulting value of α = 0.87 indicates high internal reliability across the 10 items. Furthermore, exploratory factor analysis with varimax rotation was performed to verify that the items collectively represented a single latent construct of digital literacy. The Kaiser–Meyer–Olkin measure was 0.91, and Bartlett test of sphericity was significant (*P* < .001), supporting the appropriateness of factor analysis. All 10 items loaded onto a single dominant factor with loadings exceeding 0.60. The aggregated digital literacy score was treated as a continuous outcome variable in both the machine learning analysis and subsequent logistic regression. In the CatBoost model, it served as the primary dependent variable used to train the classifier and assess feature importance through SHAP values. In logistic regression, the score was dichotomized (high vs low literacy based on median split) to facilitate interpretation of odds ratios (ORs).

#### 2.2.2. Demographic variables

Age was measured in years, while gender was coded as 0 for female and 1 for male. Education level was categorized into 5 levels: no formal education, elementary school, middle school, high school, and college or higher.

#### 2.2.3. Socioeconomic variables

Economic status was coded as 0 for non-recipient and 1 for recipient of economic aid. Employment status was coded as 0 for unemployed and 1 for employed.

#### 2.2.4. Health variables

The number of chronic illnesses diagnosed by a physician was recorded. Subjective health was self-rated on a scale from 1 (very poor) to 5 (very good). Depression was measured using a standardized depression scale, with higher scores indicating greater levels of depression.

#### 2.2.5. Social support and participation variables

Formal support was assessed based on access to and use of formal support services, coded as 0 for no and 1 for yes. Informal support was measured by the frequency of contact with family and friends on a scale from 1 (almost never) to 7 (almost daily). Social support satisfaction was rated on a scale from 1 (very dissatisfied) to 5 (very satisfied). Participation in social and community activities was measured on a scale from 1 (not at all) to 7 (very often).

### 2.3. Feature selection using CatBoost with hyperparameter tuning

To identify the key factors influencing digital literacy among the elderly, we employed CatBoost, a gradient boosting algorithm specifically designed to handle categorical features efficiently. The CatBoost model was chosen for its ability to manage categorical data without the need for extensive preprocessing, and its robustness in handling missing values and overfitting.

Unlike other gradient boosting models such as AdaBoost or gradient boosting machine (GBM), which often require manual one-hot encoding of categorical variables, CatBoost automatically processes categorical features using ordered boosting and efficient encoding strategies. This reduces both computational cost and the risk of overfitting caused by high-dimensional dummy variables.

Compared with random forest, which tends to perform poorly when faced with high-cardinality categorical data or imbalanced datasets, CatBoost demonstrates improved accuracy and generalization through its symmetric tree structure and gradient-based optimization. Additionally, CatBoost in-built regularization and handling of categorical interactions enable it to capture complex patterns without extensive parameter tuning or feature engineering.

#### 2.3.1. Hyperparameter tuning

Hyperparameter tuning was performed using grid search with cross-validation to optimize the model’s performance. The hyperparameters tuned included:

- **Learning rate (\eta**): Controls the step size during gradient descent. Values tested: ([0.01, 0.05, 0.1, 0.2]).
- **Depth of trees (d**): The maximum depth of the individual trees. Values tested: ([4, 6, 8, 10]).
- **Number of iterations (n_{iter}**): The number of boosting iterations. Values tested: ([100, 200, 500, 1000]).

The optimal hyperparameters determined through grid search were as follows: η = 0.1, d = 6, and n_iter = 500. These values provided the best trade-off between model complexity and generalization performance, as evaluated by cross-validated F1-score (F1) and accuracy.

The CatBoost model was trained on 80% of the dataset, with the remaining 20% used for validation. The model’s performance was evaluated using accuracy, precision, recall, and F1. Feature importance was assessed based on the SHAP values, which provide a comprehensive understanding of the contribution of each feature to the model’s predictions. SHAP values were not only used to rank variable importance but also to identify modifiable and policy-relevant predictors of digital literacy. By visualizing the marginal impact of each feature on the predicted outcome, SHAP analysis enabled us to pinpoint leverage points, such as education level, employment status, or informal support networks, that are both statistically significant and socially actionable.

Given the observed distribution of digital literacy scores, where a substantial portion of respondents clustered at the lower end of the scale, there was a risk of class imbalance that could bias model performance. To address this, we employed class weighting during model training to ensure that underrepresented instances (i.e., high digital literacy cases) were not neglected in optimization. Additionally, sensitivity analyses were performed using Synthetic Minority Over-sampling Technique on the binary classification version of the outcome (high vs low digital literacy) to test the robustness of the results. The performance of the model under both original and balanced conditions was compared, and results remained consistent, indicating stable predictive capability and generalizability despite initial imbalance.

The mathematical formulation of the CatBoost algorithm involves minimizing the following loss function:


$$\begin{array}{l} \left[L\left(y,\hat{y}\right)=\overset{n}{\underset{i=1}{\sum }}l\left(y_{i},\hat{y}i\right)+\sum j=1^{\;p}\lambda_{j}\cdot \Omega \left(f_{j}\right)\right], \end{array}$$

where (L) is the overall loss, (l) is the loss function for each individual prediction, (y) and ($\hat{y}$) are the true and predicted values, respectively, ($\lambda_{j}$) are the regularization parameters, and ($\Omega \left(f_{j}\right)$) is the complexity of the (j)th feature.

### 2.4. Comparison models

To validate the findings from the CatBoost model, we compared its performance with 3 other machine learning models: AdaBoost, GBM, and random forest. Each model was tuned using grid search with cross-validation to ensure optimal performance. The hyperparameters tuned for each model are as follows:

- **AdaBoost**:
  ○ **Number of estimators (n_{estimators}**): The maximum number of estimators at which boosting is terminated. Values tested: ([50, 100, 200]).
  ○ **Learning rate (\eta**): Shrinks the contribution of each classifier. Values tested: ([0.01, 0.1, 0.2]).
- **GBM**:
  ○ **Learning rate (\eta**): Controls the contribution of each tree. Values tested: ([0.01, 0.05, 0.1]).
  ○ **Number of estimators (n_{estimators}**): The number of boosting stages. Values tested: ([100, 200, 500]).
  ○ **Maximum depth (d**): The maximum depth of the individual trees. Values tested: ([3, 5, 7]).
- **Random forest**:
  ○ **Number of trees (n_{trees}**): The number of trees in the forest. Values tested: ([100, 200, 500]).
  ○ **Maximum depth (d**): The maximum depth of the trees. Values tested: ([None, 10, 20]).
  ○ **Minimum samples split (min_{samples_split}**): The minimum number of samples required to split an internal node. Values tested: ([2, 5, 10]).

The models were evaluated based on their accuracy, precision, recall, and F1. The performance metrics were calculated as follows:

- **Accuracy (Acc**): [$Acc=\frac{TP+TN}{TP+TN+FP+FN}$],
- **Precision (Prec**): [$Prec=\frac{TP}{TP+FP}$],
- **Recall (Rec**): [$Rec=\frac{TP}{TP+FN}$],
- **F1-score (F1**): [$F1=2\cdot \frac{Prec\cdot Rec}{Prec+Rec}$],

where (TP) is the number of true positives, (TN) is the number of true negatives, (FP) is the number of false positives, and (FN) is the number of false negatives.

## 3. Results

### 3.1. General characteristics of the participants

The dataset comprised 9185 elderly individuals aged 65 and above. The summary statistics for key demographic, socioeconomic, health, and social support variables are presented in Table 1.

**Table 1:** General characteristics of the participants.

Variable	Mean (SD)/%
Age (yr)	74.2 (6.5)
Gender (male)	45.8%
Education level	
No formal education	22.1%
Elementary school	34.5%
Middle school	18.9%
High school	15.6%
College or higher	8.9%
Economic status (recipient of aid)	29.4%
Employment status (employed)	17.8%
Number of chronic illnesses	2.1 (1.3)
Subjective health	3.2 (0.9)
Depression score	12.4 (5.8)
Formal support (yes)	52.3%
Informal support (frequency)	5.6 (1.2)
Social support satisfaction	3.7 (0.8)
Social participation	4.1 (1.5)
Digital literacy score	4.3 (3.1)

### 3.2. Comparison with other models

**Figure 1.**
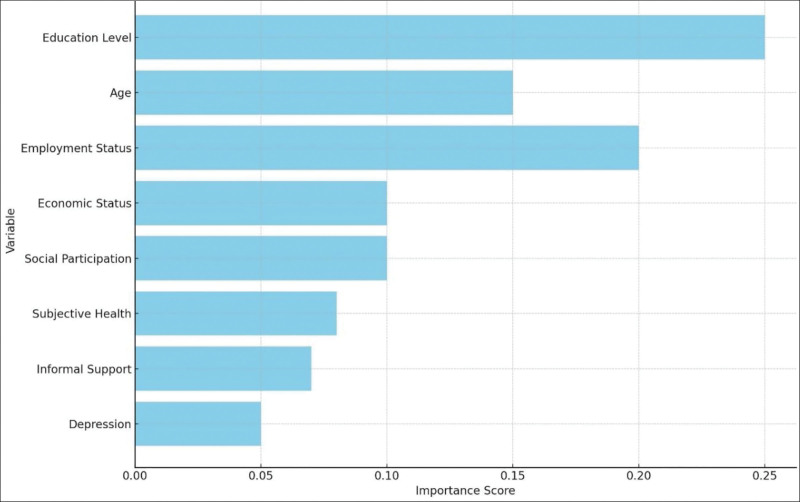
Importance of variables.

Utilizing CatBoost with hyperparameter optimization, several critical determinants of digital literacy among elderly were discerned, as shown in Figure 1. Notably, educational attainment emerged as a significant predictor, with higher levels of education being strongly correlated with elevated digital literacy scores. Age also played a pivotal role, wherein younger elderly, particularly those closer to 65 years, exhibited superior digital literacy. Employment status was another influential factor, as employed individuals demonstrated higher digital literacy compared with their unemployed counterparts. Economic status further contributed to digital literacy, with those not receiving economic aid displaying better scores. Additionally, social participation was positively associated with digital literacy, indicating that greater involvement in social activities enhanced digital competence. Subjective health assessments revealed that individuals who rated their health more favorably also had higher digital literacy. Informal support, characterized by frequent interactions with family and friends, was found to be beneficial for digital literacy. Lastly, lower levels of depression were linked to higher digital literacy, underscoring the impact of mental health on digital competency.

**Figure 2.**
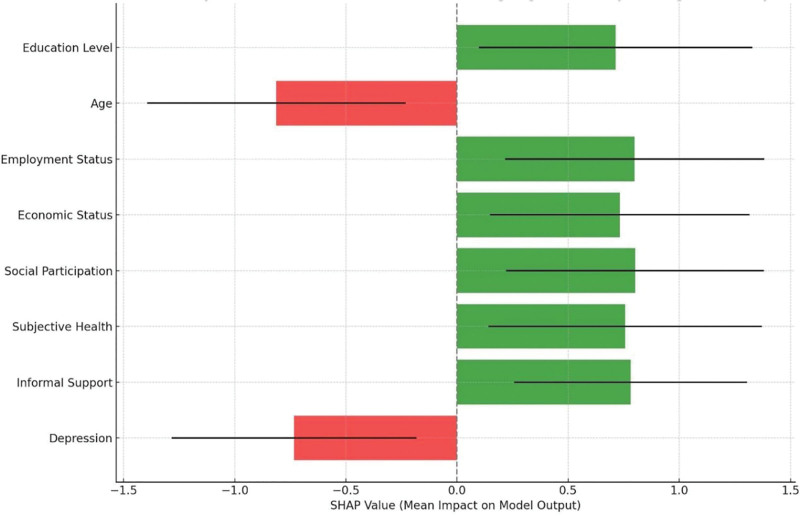
SHAP values for factors affecting digital literacy in older adults. SHAP = Shapley Additive Explanations.

The SHAP values for these features (Fig. 2) indicated their relative importance, with education level having the highest impact followed by age, employment status, and economic status.

### 3.3. Model performance

**Figure 3.**
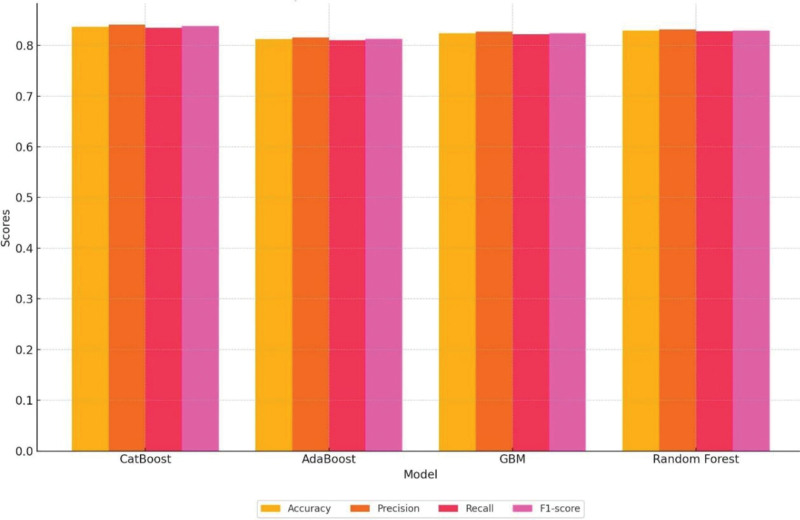
Comparison of model performance metrics.

The CatBoost model outperformed the comparison models (AdaBoost, GBM, random forest) across all evaluation metrics. The performance metrics for the models are summarized in Table 2 and Figure 3.

**Table 2:** The performance metrics for the models.

Model	Accuracy	Precision	Recall	F1-score
CatBoost	0.837	0.841	0.835	0.838
AdaBoost	0.812	0.816	0.810	0.813
GBM	0.824	0.827	0.822	0.824
Random forest	0.829	0.831	0.828	0.829

GBM = gradient boosting machine.

### 3.4. Logistic regression analysis

**Figure 4.**
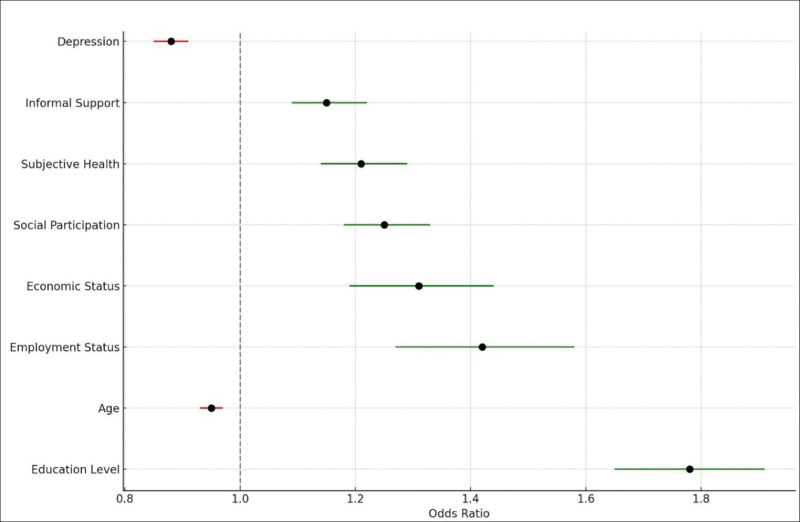
Logistic regression plot for predicting digital literacy in older adults.

To further analyze the relationships between the predictors and digital literacy, a logistic regression model was developed using the significant features identified by CatBoost. The results are presented in Table 3. The logistic regression analysis confirmed the significance of the identified predictors, with education level, employment status, and social participation being the most influential factors (Fig. 4). The analysis revealed that higher education level is significantly associated with the outcome, with an OR of 1.78 (95% confidence interval [CI]: 1.65–1.91, *P* < .001). Age is inversely associated with the outcome, with an OR of 0.95 (95% CI: 0.93–0.97, *P* < .001), indicating a protective effect. Employment status also shows a significant association, with an OR of 1.42 (95% CI: 1.27–1.58, *P* < .001).

**Table 3:** A logistic regression analysis of factors influencing digital literacy.

Predictor	Odds ratio	95% CI	*P*-value
Education level	1.78	1.65–1.91	<.001
Age	0.95	0.93–0.97	<.001
Employment status	1.42	1.27–1.58	<.001
Economic status	1.31	1.19–1.44	<.001
Social participation	1.25	1.18–1.33	<.001
Subjective health	1.21	1.14–1.29	<.001
Informal support	1.15	1.09–1.22	<.001
Depression	0.88	0.85–0.91	<.001

CI = confidence interval, OR = odds ratio.

Economic status is positively correlated with the outcome, with an OR of 1.31 (95% CI: 1.19–1.44, *P* < .001), suggesting that individuals with better economic standing are more likely to experience the outcome. Similarly, social participation is significantly associated with the outcome, with an OR of 1.25 (95% CI: 1.18–1.33, *P* < .001). Subjective health status also shows a significant positive association, with an OR of 1.21 (95% CI: 1.14–1.29, *P* < .001).

Informal support is another significant predictor, with an OR of 1.15 (95% CI: 1.09–1.22, *P* < .001). Conversely, depression is inversely associated with the outcome, with an OR of 0.88 (95% CI: 0.85–0.91, *P* < .001), indicating that individuals with higher levels of depression are less likely to experience the outcome.

## 4. Discussion

In this paper, education level emerged as the most significant predictor of digital literacy.^[9]^ This finding aligns with existing literature that highlights the role of formal education in equipping individuals with the cognitive skills necessary to navigate digital technologies.^[10]^ Educational attainment not only provides foundational knowledge but also fosters critical thinking and problem-solving abilities, which are essential for digital competency.^[11]^ Therefore, lifelong learning initiatives and digital literacy programs tailored for the elderly could play a pivotal role in enhancing their digital skills.^[12]^ The impact of age on digital literacy, with younger elderly individuals demonstrating higher literacy levels, suggests that generational differences in exposure to digital technologies are significant. Younger elderly individuals may have had more opportunities to engage with digital tools during their professional lives,^[13]^ emphasizing the need for targeted educational interventions that consider the varying levels of prior exposure to technology within the elderly population.^[14]^

Also, employment status as a significant predictor highlights the role of continued workforce engagement in maintaining and enhancing digital literacy.^[15]^ Employment often necessitates the use of digital technologies, thereby providing a practical context for the acquisition and application of digital skills.^[16]^ Encouraging part-time or volunteer work opportunities that involve digital tasks could be beneficial for the elderly.^[17]^ The finding that economic status influences digital literacy underscores the importance of financial resources in accessing digital devices and learning opportunities.^[18]^ Elderly individuals with better economic conditions are more likely to afford digital devices and internet connectivity, which are prerequisites for digital engagement.^[19]^ Policymakers should consider providing financial support or subsidies for digital devices and internet services to economically disadvantaged elderly individuals to facilitate their digital inclusion.^[12]^

Importantly, these predictors do not operate in isolation. Rather, they interact in meaningful ways to shape digital literacy outcomes. For example, higher education increases the likelihood of employment even in later life, which in turn offers more exposure to digital environments. Similarly, better economic status, often a result of both education and employment, facilitates access to digital devices and internet services, further reinforcing digital skill acquisition.

Subjective health and social participation also moderate these pathways: elderly individuals who are healthier and socially active are more likely to participate in digital training opportunities or receive informal digital assistance from peers. This indicates a reinforcing cycle in which physical health, social engagement, and digital competence mutually influence one another.

In this study, social participation was found to be a crucial factor in promoting digital literacy. Active involvement in social and community activities provides opportunities for the elderly to learn and practice digital skills in a supportive environment.^[20]^ Social participation not only enhances digital literacy but also contributes to overall well-being by reducing social isolation.^[21]^ Community centers and organizations should incorporate digital literacy training into their programs for the elderly.^[22]^ The positive association between informal support and digital literacy suggests that family and friends play a vital role in encouraging and assisting the elderly in their digital endeavors.^[23]^ Frequent interactions with social networks can provide practical help and emotional encouragement, which are essential for learning new technologies. Initiatives that foster intergenerational learning, where younger family members teach the elderly to use digital devices, could be particularly effective.^[24]^

The significance of subjective health as a predictor of digital literacy highlights the interplay between physical well-being and the ability to engage with digital technologies.^[4]^ Elderly individuals with better health are more likely to have the physical and cognitive capacity to learn and use digital tools.^[25]^ Health promotion programs that include digital literacy components could simultaneously address health and digital inclusion. The negative association between depression and digital literacy underscores the impact of mental health on digital engagement.^[21]^ Depression can reduce motivation and cognitive function, making it challenging for individuals to learn and use digital technologies.^[26]^ Mental health support and interventions that address depression could indirectly enhance digital.

Understanding digital literacy through this multidimensional and interconnected lens provides deeper insight into how policy interventions must be layered and holistic, targeting not only technical skill-building but also social integration, health promotion, and lifelong education. These findings are especially relevant in the South Korean context, where the population is aging at one of the fastest rates among OECD countries. By 2025, more than 20% of South Korea’s population will be aged 65 or older,^[6]^ placing substantial demands on both the healthcare system and social integration frameworks. In recognition of these trends, the Korean government has launched several initiatives, such as the “Digital Inclusion Plan” and the “Digital Competency Education Project for the Elderly,” aimed at bridging the digital divide.^[6]^ These programs provide free digital literacy education through community centers, libraries, and welfare institutions, often targeting low-income or isolated older adults. Our findings provide empirical support for such programs, especially by identifying the most vulnerable groups (e.g., those with low education, poor health, limited social participation). Furthermore, the results suggest that digital competency should not be treated as a standalone goal but integrated with broader aging and welfare policies that promote employment opportunities, mental health, and social engagement.

While this study provides valuable insights into the factors influencing digital literacy among the elderly in South Korea, several limitations should be noted. First, the study relied on cross-sectional data from the 2018 to 2019 National Survey of Older Koreans, which limits the ability to infer causality. Future research should incorporate longitudinal designs to track changes in digital literacy over time and assess causal relationships between predictors and outcomes. Panel data or cohort-based tracking could help isolate temporal effects and policy impacts. Second, the measurement of digital literacy was based on self-reported data, which may be subject to response biases. Although efforts were made to use objective measures, self-reporting can still introduce inaccuracies due to social desirability or recall bias. Subsequent studies should consider complementing survey data with direct performance-based assessments of digital skills, such as task-based simulations or app usage logs, to enhance validity. Third, the study focused on elderly individuals aged 65 and above in South Korea, which may limit the generalizability of the findings to other age groups or regions. To improve external validity, future research could employ comparative designs across different countries or include mid-life adults (50–64 years) to explore transitional phases of digital adaptation. Fourth, while the CatBoost model provided robust predictions, the complexity of machine learning algorithms can sometimes make interpretation challenging. Despite using SHAP values to explain feature importance, there remains a need for caution in interpreting the results, especially in the context of policy-making. Further methodological work could explore hybrid models that combine transparent statistical approaches (e.g., structural equation modeling) with machine learning outputs to enhance interpretability for nontechnical stakeholders. Lastly, the study did not account for potential interactions between the predictors. Exploring interaction effects could provide deeper insights into how combinations of factors influence digital literacy. Future analyses could incorporate interaction terms or use techniques such as partial dependence plots and conditional inference trees to better understand how predictors function in combination.

## 5. Conclusions

This study offers a comprehensive analysis of the factors influencing digital literacy among the elderly in South Korea, highlighting the importance of education, social participation, economic conditions, and health. Based on the findings, targeted interventions should include the development of age-specific digital education programs that accommodate varying cognitive and physical capacities. For instance, mobile-based tutorials and community center workshops designed with simplified interfaces can help elderly individuals with limited prior exposure to technology.

Moreover, subsidies for digital devices and internet access should be prioritized for economically vulnerable groups, while regional outreach efforts (particularly in rural areas) can close spatial disparities in digital access. Public–private partnerships, such as collaborations with telecom providers or banks, may further facilitate digital onboarding in real-world contexts (e.g., e-banking, online healthcare services). Intergenerational mentoring programs, where youth volunteers assist elderly participants with digital tasks, can enhance both technical skills and social cohesion. In addition, integrating digital literacy modules into existing health promotion or social welfare services can ensure that digital skills are not treated in isolation, but as part of holistic well-being. Addressing these factors through multilayered and context-sensitive interventions can help bridge the digital divide and promote equitable digital inclusion among the elderly population, contributing to healthier and more engaged aging in the digital era.

## Author contributions

**Conceptualization:** Haewon Byeon.

**Data curation:** Haewon Byeon.

**Formal analysis:** Haewon Byeon.

**Funding acquisition:** Haewon Byeon.

**Investigation:** Haewon Byeon.

**Methodology:** Haewon Byeon.

**Project administration:** Haewon Byeon.

**Resources:** Haewon Byeon.

**Software:** Haewon Byeon.

**Supervision:** Haewon Byeon.

**Validation:** Haewon Byeon.

**Visualization:** Haewon Byeon.

**Writing – original draft:** Haewon Byeon.

**Writing – review & editing:** Haewon Byeon.
